# Anti-Hypolipidemic and Anti-Oxidative Effects of Hydroalcoholic Extract of *Origanum majorana* on the Hepatosteatosis Induced with High-Fat Diet in Rats

**DOI:** 10.21315/mjms2020.27.1.6

**Published:** 2020-02-27

**Authors:** Abdolmomen Ghaeni Pasavei, Reza Mohebbati, Nadia Boroumand, Ahmad Ghorbani, Azar Hosseini, Shirin Taraz Jamshidi, Mohammad Soukhtanloo

**Affiliations:** 1 Department of Clinical Biochemistry, Faculty of Medicine, Mashhad University of Medical Sciences, Mashhad, Iran; 2 Department of Physiology, Faculty of Medicine, Mashhad University of Medical Sciences, Mashhad, Iran; 3 Pharmacological Research Center of Medicinal Plants, Mashhad University of Medical Sciences, Mashhad, Iran; 4 Department of Pathology, Faculty of Medicine, Mashhad University of Medical Sciences Mashhad, Iran

**Keywords:** non-alcoholic fatty liver disease, oxidative stress, Origanum majorana, high-fat diet

## Abstract

**Introduction:**

The aim of the current study is to evaluate the antihyperlipidemic and anti-oxidative effects of hydro-alcoholic extract of marjoram (HAEM) in rats fed with a high-fat diet (HFD).

**Methods:**

In the experimental study, the rats were randomly divided into four groups of five rats in each and fed with high-fat diet for 12 weeks as follows: One group (normal diet group) was fed with a standard diet, one group was fed with HFD, and two groups were fed with HFD and orally fed with 150 and 450 mg/kg/day HAEM. The serum samples and liver tissues were used for measuring the biochemical and oxidative parameters and histopathological studies. HFD induced hepatosteatosis in rats as evidenced by the altered liver enzymes activity, serum lipid profile and oxidative status.

**Results:**

Serum lipid profile (triglyceride, cholesterol and low-density lipoprotein) in rats fed with HFD + HAEM (150 and 450 mg/kg/day) was significantly decreased. Furthermore, the evaluation of oxidative stress showed a reduction of the malondialdehyde (MDA) level and an increase in ferric-reducing anti-oxidant power. Meanwhile, liver enzyme activities declined in response to HAEM.

**Conclusion:**

Using the HAEM could be a future therapeutic agent in treating hepatosteatosis and reducing oxidative damages of HFD in the liver.

## Introduction

Non-alcoholic fatty liver disease (NAFLD), characterised by the triglycerides and cholesterol accumulation within the hepatocytes, has been identified as one of the major causes of liver diseases (i.e., fibrosis, cirrhosis and hepatocellular) in the world (1). Studies have shown that diet has a significant role in the pathogenesis of this disease. It is proven that the high-fat diet (HFD) is one of the risk factors for NAFLD acting through inducing oxidative stress (2, 3). The altered redox balance is suggested to be involved in the occurrence of steatosis, fibrosis, and steatohepatitis (4).

The existence of oxidative stress in NAFLD can be explained by the role of mitochondria in hepatic lipid metabolism. Mitochondria are known with the ability to adapt the rate of beta-oxidation based on the amount of lipid accumulation in hepatocytes. However, following the elevated delivery of substrates to the mitochondria, the generation of reactive oxygen species (ROS) increases that eventually may result in mitochondria dysfunction. Depletion in the rate of mitochondria function along with the high rate of beta-oxidation causes generation of incomplete oxidation products and increased ROS, all of which could be involved in NAFLD pathogenesis (5).

The altered redox status could lead to hepatic damage by causing alteration of lipids, proteins, and DNA contents. It also could affect pathways that modulate normal biological functions such as cell apoptosis and hepatic stellate cell activation pathways. Therefore, oxidative stress followed by lipid peroxidation and inflammation could be responsible for the disease progression and alteration in antioxidant enzyme status (6–8).

Regarding the important role of oxidative stress in NAFLD pathogenesis, the effectiveness of several anti-oxidants has been examined in treating NAFLD. Using natural anti-oxidants has shown clear benefits in the biochemical and histological improvement of NAFLD. These anti-oxidants often provide potential free-radical scavenging activities along with anti-inflammatory abilities, which are crucial factors in NAFLD treatment (7–9). It has been demonstrated that many edible or medicinal plants such as green tea (10), *Curcuma longa* (11), *Nigella sativa* (12) and marjoram oil (13) could protect the liver from oxidative damage through their anti-oxidant activities. Furthermore, *Origanum majorana* (known as sweet marjoram) possesses potential anti-oxidant capacity mainly because of its polyphenolic compounds. Although this herb has been cultivated in several countries, it is known to be native to Mediterranean region. *Origanum majorana* is a perennial bushy herb that grows up to 30 cm–60 cm. Not only this plant has been used for garnishing and flavouring but also it has shown to be advantageous in traditional and herbal medicine. Therefore, different compounds have been identified in this herb, each having its own special pharmacological activity. To date, 31 polyphenols have been reported in marjoram. Among them, Rosmarinic acid was identified to be the strongest anti-oxidant polyphenol mostly responsible for the anti-oxidant activity of marjoram (14–16). The potential anti-oxidant capacity of marjoram and its protective activity against liver damage has been further proved by other studies (17).

Despite the discussed studies about anti-oxidants activity of marjoram, to the best of authors’ knowledge, the in-vivo studies regarding hepatoprotective properties of this natural compound are limited. Hence, the aim of the current study was to evaluate the anti-oxidant activity and hepatoprotective properties of hydro-alcoholic extract of marjoram (HAEM) in non-alcoholic fatty liver induced by HFD in rats. To this end, the effects of marjoram treatment on the status of malondialdehyde (MDA), thiol groups, catalase, superoxide dismutase and myeloperoxidase were evaluated. The hepatoprotective activity of marjoram was further assessed by lipid profile and liver tests including aspartate aminotransferase (AST), alanine aminotransferase (ALT), gamma-glutamyl transferase (GGT), alkaline phosphatase (ALP) and bilirubin.

## Materials and Methods

### Animals

A total of 20 six-week-old male Wistar rats with a body weight of 200 ± 10 g (Animal House, School of Medicine, Mashhad University of Medical Sciences and Mashhad, Iran) were tested in this study. They were housed at 23 ± 1 °C with 12 h light/dark cycles with free access to a standard diet and tap water.

### Preparation of 70% Ethanolic Extract of Marjoram

The *Origanum majorana* were purchased from Imam Reza Pharmacy (Division of Medicinal Plants, Mashhad, Iran) and identified by Eng. Joharchi in the herbarium of Ferdowsi University of Mashhad). A voucher specimen for considered herb is 17987.

*Origanum majorana* samples were powdered and then 200 g of dry powder of *marjoram* leaves was stirred in 1200 mL 70% (w/v) ethanol and shaken for 72 h at 37 °C. The mixture was filtered through 1 mm and 0.1 mm filters, in the order of their appearance. The solvent evaporation process was carried out at 37 °C. The final dried extract was kept at −20 °C (18). The ethanol was evaporated and the extract was concentrated to the desired level and stored under −20 °C until use.

## Experimental Design

In the experimental study, the rats were randomly divided into the following four groups: i) normal control (NC) group; ii) HFD group; iii) HFD + HAEM (150 mg/kg/day) group; and iv) HFD + HAEM (450 mg/kg/day) group. The number of rats was the same (*n* = 6) in each group. To investigate the protective activity of HAEM against NAFLD induced by HFD, marjoram extract (150 or 450 mg/kg/day) and/or HFD were administered to the animals daily for 12 consecutive weeks. At the end of the experiment, the rats were sacrificed after 12 consecutive weeks of administering their respective diets and 2 cc of their blood was taken for biochemical analysis. Following blood collection, the liver was excised and rinsed in phosphate-buffered saline (PBS). For histochemical analysis, the obtained sections were placed in 10% formalin. The induction of steatosis in rat through an HFD was assessed by microscopic evaluation of hematoxylin and eosin (H&E) stained sections. The remaining tissues were utilised for tissue homogenate to perform anti-oxidant assays.

Next, the liver (0.2 g) was scissor-minced, washed with normal saline and homogenised in ice solution containing 2 mL of 0.2 M phosphate buffer (pH 7) with a homogeniser device (T 18 B-Laboratory equipment, Germany). Homogenates were centrifuged at 1100 g for 10 min at 4 °C to remove tissue remnants. The supernatant was restored at −70 °C until measuring the parameters. The samples were analysed using the double-blinded method.

### Ferric-Reducing Anti-Oxidant Power Assay

The ferric reducing anti-oxidant power (FRAP) assay determines the potency of anti-oxidants in reducing the Fe^3+^ to the blue coloured Fe^2+^ form in 2,4,6-tripyridyl-s-triazine (TPTZ) complex. So, the FRAP reagent was prepared by mixing 100 mL of phosphate buffer, 10 mL of TPTZ solution, 10 mL of FeCl_3_ and 12 mL of distilled water at 37 °C in a water bath. The assay was done according to the standard procedure (19). Briefly, 1 mL of FRAP reagent was added to 30 μL of the homogenate test samples, the standards or the distilled water as a blank. Following 4 min incubation at 37 °C, the absorbance of the samples was read by spectrophotometer at 595 nm.

### Biochemical Markers Assay

Hepatotoxicity activity of hepatic enzymes (i.e., ALT, AST and ALP) was assessed using a commercial kit from Pars Azmoon (Iran). The activity of GGT was determined using a kit from Biosystems (Iran). The levels of serum total cholesterol, triglyceride (TG) and low-density lipoprotein (LDL) were evaluated using the Bionic kit (France). The level of bilirubin (total and direct) was determined using a commercial colorimetric kit from Pars Azmoon (Iran). Lipid peroxidation was expressed as MDA content by determining thiobarbituric acid reactive substances (TBARS) through a colorimetric method as described by Satoh (20).

### Anti-Oxidant Enzymes Assay

The tissue homogenate was also utilised to determine the anti-oxidant status. For this purpose, the activity of catalase (CAT), superoxide dismutase (SOD), thiol groups and myeloperoxidase (MPO) were measured in rats liver homogenate. The CAT activity was determined according to the method described by Aebi (21). This method is based on the decomposition of H_2_O_2_ to water-mediated by CAT. This reaction is along with the reduction of absorbance at 420 nm. The SOD activity was measured as described by Nishikimi et al. (22). The principle of this method is based on the ability of SOD to inhibit the reaction of 3-(4,5-dimethylthiazol-2-yl)-2,5-diphenyltetrazolium bromide (MTT) with superoxide anion produced through autoxidation of pyrogallol. Thiol groups were measured according to the method described by Hu and Dillard (23). In this colorimetric method, the Ellman’s reagent (5,5′-dithio-bis-[2-nitrobenzoic acid] or DTNB) reacts with sulfhydryl groups to form a complex. This reaction yields a colored product with maximum absorption at 412 nm. The activity of myeloperoxidase was measured according to the method described elsewhere (24).

## Histopathological Examination

Tissue specimens removed from animals were preserved in 10% neutral buffered formalin. Briefly, the formalin-fixed tissues were dehydrated in different grades of alcohol and cleared in xylene. Subsequently, the samples were embedded in paraffin and 5 μ-thick sections were cut by a microtome. Finally, the prepared sections were stained with H&E according to Bancroft et al. (25).

### Statistical Analysis

The SPSS 16 software package (SPSS Inc., Illinois) was used in all the statistical analyses conducted in this study. Data were expressed as mean (± standard deviation). The data normalisation was investigated by the Kolmogorov-Smirnov test. Statistical analysis was performed using a one-way analysis of variance (ANOVA) followed by Dunnett’s two-sided post hoc test for multiple comparisons. The *P* < 0.05 was considered to be statistically significant.

## Results

### Induction of Steatosis in Rat through a High Fat Diet

Rats were sacrificed after 12 consecutive weeks of administering their respective diets. Liver steatosis was assessed by microscopic evaluation of H&E stained sections.

**Figure 1:**
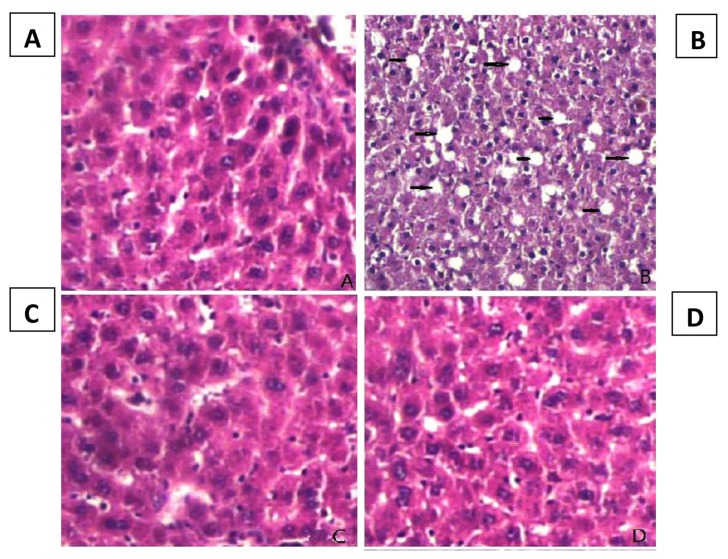
H&E stained sections of the sacrificed rats’ liver at at 400× magnification. (A) Control group with normal hepatic tissue, (B) HFD group with vacuolation in hepatocytes presented the steatosis, (C) HFD + HAEM group (150 mg/kg/day) presented normal hepatic tissue, (D) HFD + HAEM group (450 mg/kg/day) presented normal hepatic tissue

The stained sections demonstrated the increasing degree of steatosis in rats with HFD evident by the vacuolation in hepatocytes (Figure 1). However, both HFD + HAEM (150 mg/kg/day) and HFD + HAEM (450 mg/kg/day) groups showed a pattern similar to that of the NC group.

### Anti-Oxidant Enzymes

**Figure 2:**
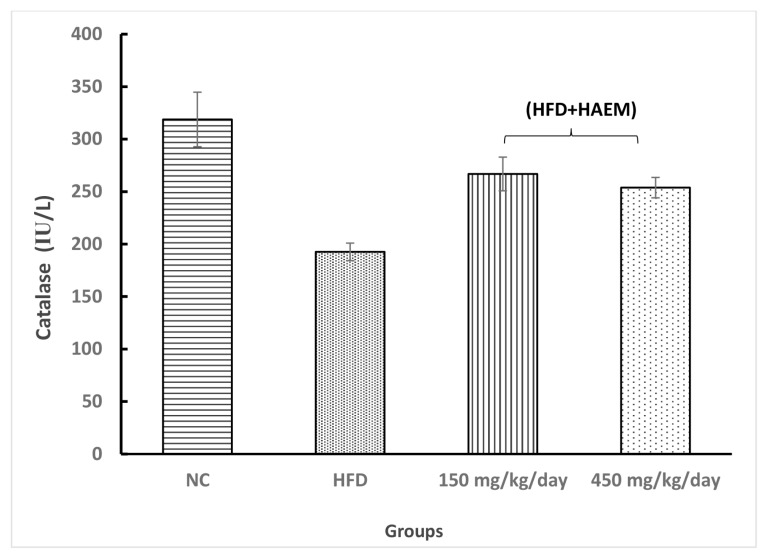
Effect of HAEM on catalase activity in rats liver homogenate

**Figure 3:**
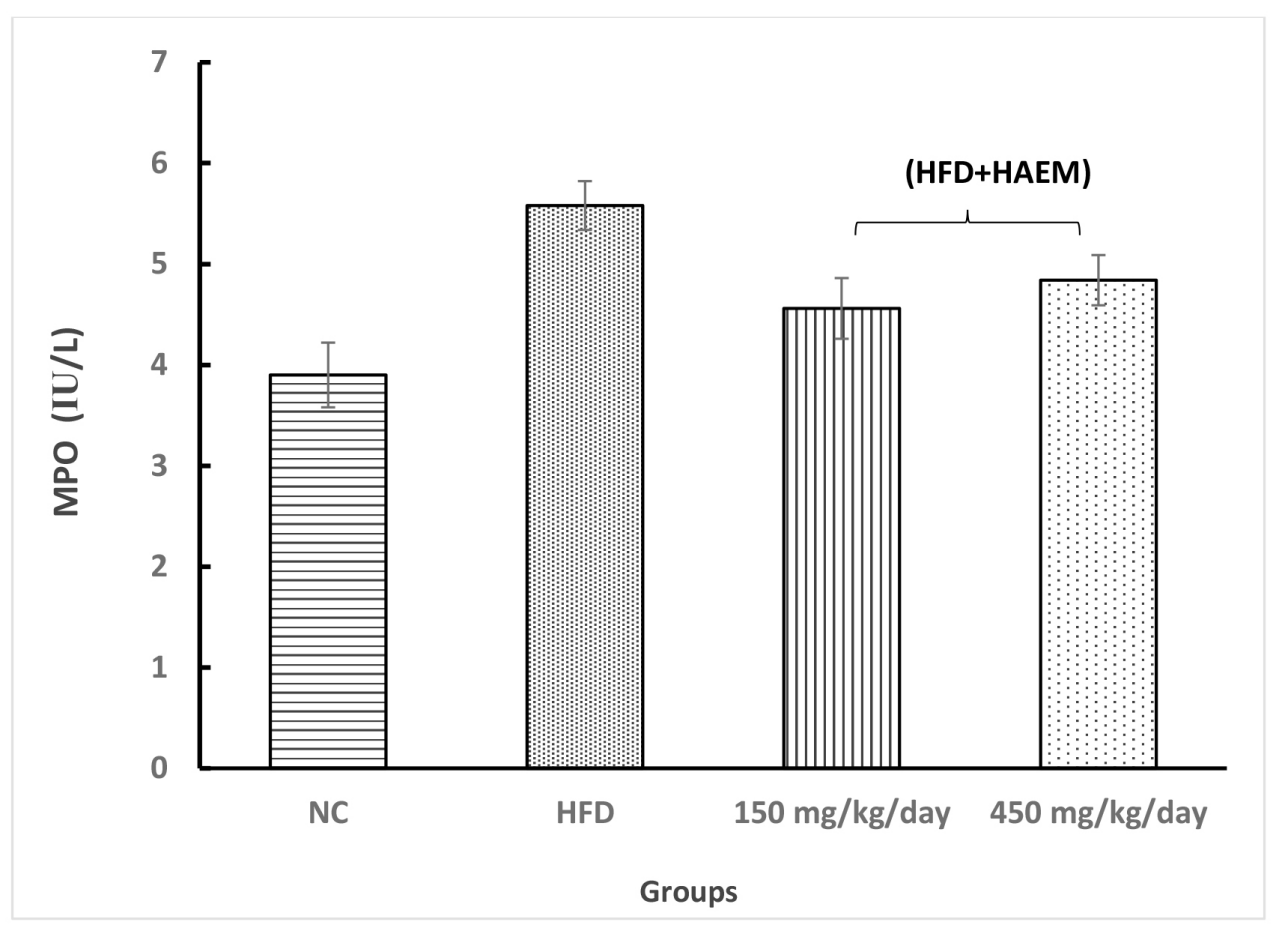
Effect of HAEM on myeloproxidase activity in rats liver homogenate

**Figure 4:**
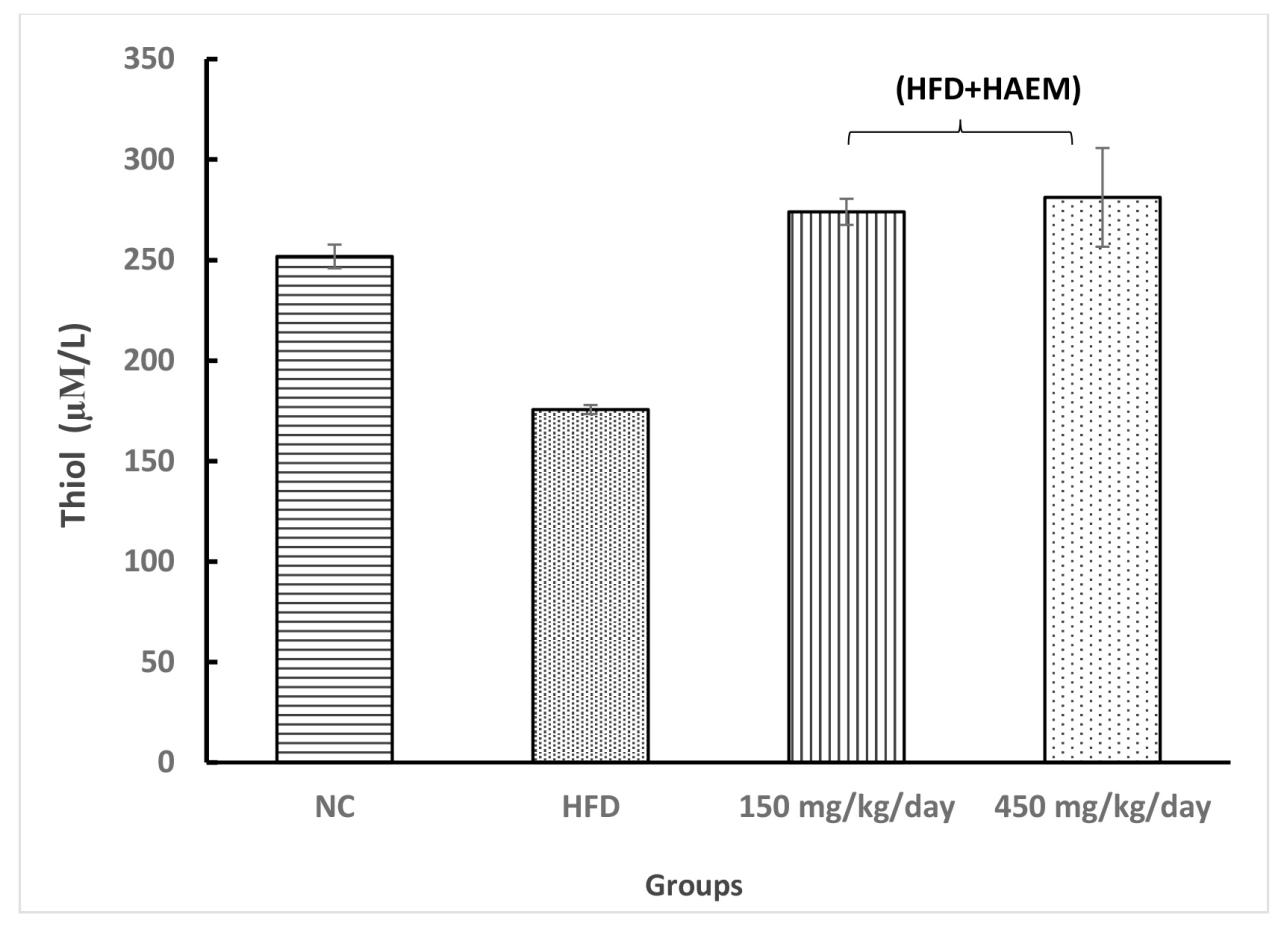
Effect of HAEM on Thiol content in rats liver homogenate

**Figure 5:**
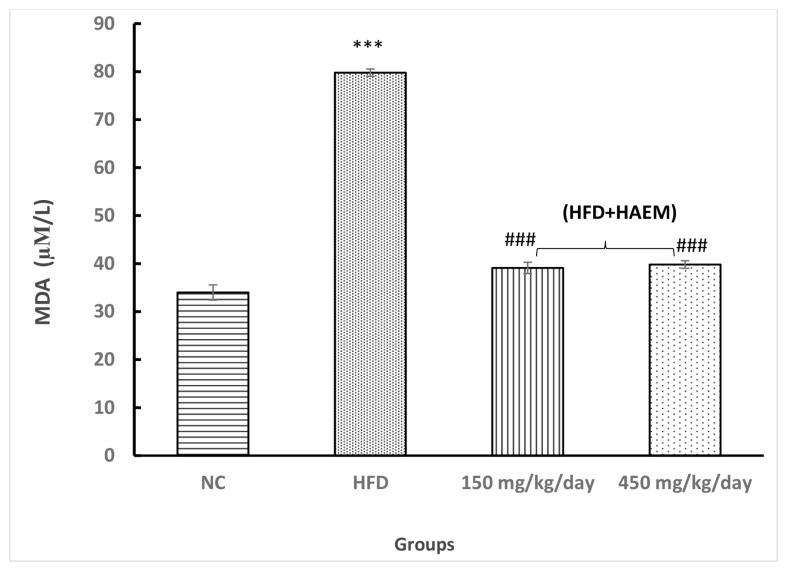
Effect of HAEM on malondialdehyde concentration in rats liver homogenate Notes: ***P < 0.001 compared to control; ###*P* < 0.001 compared to HFD-treated group

**Figure 6:**
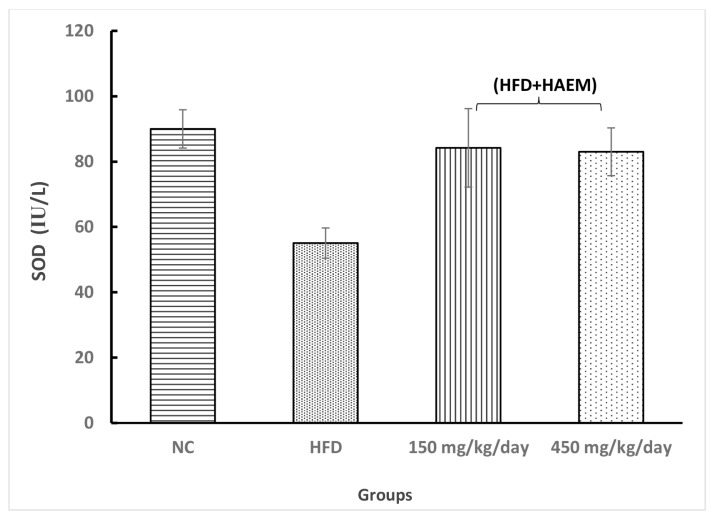
Effect of HAEM on SOD activity in rats liver homogenate

Assessing thiol groups and SOD and CAT status presented increased levels in both treatment groups; however, it was not statistically significant. Interestingly, the amount of thiol groups was elevated after treatment as compared with the control group. Nevertheless, the SOD and CAT levels did not reach the control levels after the treatment. Rats treated with marjoram at doses 150 mg/kg/day and 450 mg/kg/day showed a significant decline in MDA levels. However, the levels of MDA did not reach NC levels. The levels of MPO decreased quite similarly in both treatment groups but it did not reach the statistical significance (Figures 2–6).

### FRAP Assay

**Figure 7:**
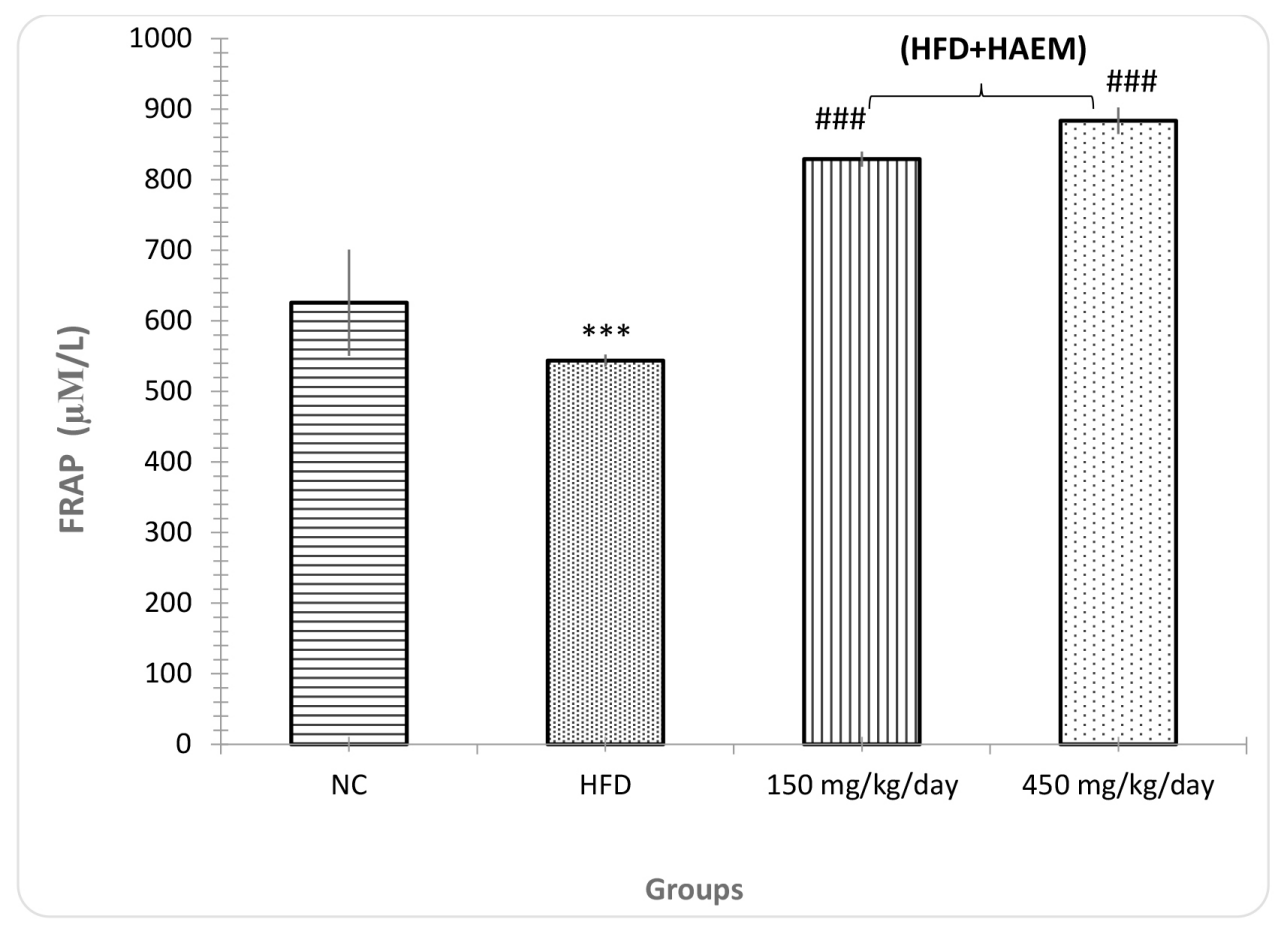
Effect of HAEM on FRAP (anti-oxidant power test) in rats liver homogenate Notes: ***P < 0.001 compared to control; ###*P* < 0.001 compared to HFD-treated group group

FRAP was significantly different between both treatment groups and HFD. As shown in the chart, the HFD group presented lower anti-oxidant capacity in comparison with the control group (*P* < 0.001), as expected. Moreover, both treatment groups (150 mg/kg/day and 450 mg/kg/day) showed significantly higher anti-oxidant capacity as compared with the HFD group (*P* < 0.001) (Figure 7).

### Lipid Profile and LDL

As expected, the HFD presented higher levels of TG as compared with the control group (*P* < 0.05). Also, both treatment groups (150 mg/kg/day and 450 mg/kg/day) demonstrated that marjoram treatment significantly reduced the levels of TG in NFALD rats (*P* < 0.001).

The chart shows that cholesterol levels increased in the HFD group compared to the control group, although statistically nonsignificant. Nevertheless, compared with the HFD group, the levels of cholesterol were lower in one treatment group (450 mg/kg/day) but not the other one (150 mg/kg/day).

The levels of LDL were different between both treatment groups and the HFD group. However, the low dosage of marjoram (150 mg/kg/day) showed a non-significant result than the high dosage (450 mg/kg/day) (Table 1).

### Hepatic Enzyme and Bilirubin

The levels of ALT and AST notably decreased in both treatment groups as compared with the HFD group. However, the ALT and AST levels did not reach the control levels after treatment.

Similarly, ALP concentration, compared with the HFD group, showed a remarkable difference in both treatment groups (*P* < 0.001). Furthermore, the ALP levels reach control levels after treatment. The difference between the HFD group and control groups was statistically significant (*P* < 0.001).

As the chart presents, the levels of GGT decreased in both treatment groups compared with the HFD group; however, the difference was not statistically significant. Also, the elevation in the HFD group did not reach the statistical significance as compared to the control group.

Direct bilirubin did not show any difference between the groups. The total bilirubin decreased slightly in both treatment groups (Table 1).

## Discussion

The current study evaluated the effects of HAEM on oxidative stress, anti-oxidant enzymes, lipid profile and liver parameters in rats fed with HFD. The obtained results presented an elevation in anti-oxidant capacity of *Origanum majorana* treated groups confirmed by FRAP, MDA, SOD, CAT and thiol groups status in the treated groups compared to the HFD group. However, the alteration in SOD, CAT, and thiol groups did not show any significant difference between the groups. In the case of lipid profile, administering *Origanum majorana* decreased the levels of TG, cholesterol and LDL. Moreover, the change in liver parameters including ALT, AST, ALP, GGT and bilirubin proved the hepatoprotective effect of *Origanum majorana*. Nevertheless, as mentioned earlier, the effect of this compound was not statistically significant on the levels of some parameters.

NAFLD has been identified as one of the major causes of liver disease in the world (1). Among various underlying factors, the diet shows a pivotal role in the pathogenesis of the disease. HFD is one of the risk factors for NAFLD, acting through inducing oxidative stress (2, 3). The altered redox balance is suggested to be involved in the occurrence of steatosis, fibrosis and steatohepatitis (4). Accordingly, we decided to induce NAFLD in rats’ liver by feeding them with HFD, which consequently resulted in induced oxidative stress and its deleterious effects. Our results demonstrate the altered redox balance in the rats fed with HFD in comparison to the control groups. Similarly, numerous studies have examined the effect of HFD on liver redox status. In this regard, Jarukamjorn et al. (26) reported an increase in the level of MDA in the liver of rats by the administration of a hypercholesterolemia diet with high sucrose for 8 weeks. Consistent with this work, the study conducted by Dhibi et al. (27) illustrated that hypercholesterolemia diet with trans fatty acids increased MDA concentration and reduced CAT and SOD activities.

The effect of oxidative stress on inducing NAFLD can be explained by the role of mitochondria in hepatic lipid metabolism. Mitochondria are known to be able to adopt the rate of beta-oxidation based on the amount of lipid accumulation in hepatocytes. However, following the elevated delivery of substrates to the mitochondria, the generation of reactive oxygen species (ROS) increases, probably leading to mitochondria dysfunction. Depletion in the rate of mitochondria function along with the high rate of beta-oxidation causes generation of incomplete oxidation products and increased ROS production is involved in NAFLD pathogenesis (5).

Regarding the important role of oxidative stress in NAFLD pathogenesis, the effectiveness of several anti-oxidants was examined in treating NAFLD. Accumulated evidence has shown the clear benefit of using natural anti-oxidants in the biochemical and histological improvement of NAFLD; as we did in this study. For instance, in a review study by Ued and Weffort (28), it was reported that adequate intake of vitamins and appropriate physical activity could significantly improve liver histology and laboratory tests. On the other hand, studies have shown that HFD results in histologic and metabolic derangements in animal models. In fact, H&E stained liver sections showed a progressive development of steatosis and a notable elevation in intrahepatic fat deposition in the HFD group (29). In this regard, the induction of NAFLD by HFD has also been evidenced by the higher liver size and weight and the percentage of lipid accumulation, all suggesting the negative effect of HFD on liver histology (30).

Regarding the histological improvement, our results demonstrated that HFD group, with vacuolation in hepatocytes, presented the steatosis while both HFD + HAEM (150 mg/kg/day) and HFD + HAEM (450 mg/kg/day) groups showed normal hepatic tissue, indicating the positive effect of natural anti-oxidant on liver histology.

Regarding the biochemical improvement, studies have shown that natural anti-oxidants often provide potential free-radical scavenging activities along with anti-inflammatory abilities, which are crucial factors in NAFLD treatment (7–9). In this regard, it has been demonstrated that many edible or medicinal plants such as green tea (10), *Ziziphus mauritiana* leaf (31), jujube honey (32), virgin olive oil (33) and marjoram oil (13) can protect the liver from oxidative damage through their anti-oxidant activities. Among various medicinal plants, *Origanum majorana* (known as sweet marjoram) has been known for its anti-oxidant capacity which is mainly because of its polyphenolic compounds. Up to now, a total of 31 polyphenols has been reported in marjoram. Among them, rosmarinic acid was identified to be the strongest anti-oxidant polyphenol mostly responsible for the anti-oxidant activity of this herb (14–16). The potential anti-oxidant capacity of marjoram and its protective activity against liver damage has been further proved by other studies (17). In accordance with this result, we observed that the rats treated with *Origanum majorana* at doses 150 mg/kg/day and 450 mg/kg/day showed a significant decline in MDA levels. However, the levels of MDA did not reach that of NC levels. Moreover, evaluation of thiol groups and SOD and CAT status presented increased levels in both treatment groups; however, it was not statistically significant probably due to the small number of samples. Interestingly, the amount of thiol groups increased after treatment more than the control group. Nevertheless, the SOD and CAT levels did not reach the control levels after the treatment. The results of the FRAP assay further supports the anti-oxidant activity of *Origanum majorana* as the treatment groups (150 mg/kg/day and 450 mg/kg/day) showed significantly higher anti-oxidant capacity compared with HFD group (*P* < 0.001).

Regarding the lipid profile, the present study demonstrated that HFD could remarkably increase the levels of TG, cholesterol and LDL as compared to the control groups. These results are compatible with those reported by Cui et al. and Yang et al. (34, 35). It is noteworthy that after the treatment with *Origanum majorana*, both treatment groups (150 mg/kg/day and 450 mg/kg/day) demonstrated remarkably reduced levels of TG (*P* < 0.001). Moreover, the levels of LDL between both treatment groups and the HFD group were significantly different. However, the low dosage of marjoram (150 mg/kg/day) showed a more significant result (*P* < 0.01) than the high dosage (*P* < 0.05) (450 mg/kg/day). Furthermore, the levels of cholesterol were significantly lower in one treatment group (450 mg/kg/day) (*P* < 0.05), but not the other one (150 mg/kg/day). Despite some minor differences between the effect of 150 mg/kg/day and 450 mg/kg/day dosages, our obtained results demonstrated the protective effect of *Origanum majorana* in vivo.

To further assess the hepatoprotective effect of *Origanum majorana*, we examined its effect on liver parameters particularly. Based on the results of this study, the levels of ALT and AST notably decreased after the treatment with 150 mg/kg/day and 450 mg/kg/day dosages of *Origanum majorana* as compared to the HFD group. However, the ALT and AST levels did not reach the control levels after the treatment. Likewise, regarding ALP, both treatment groups showed a remarkable difference (*P* < 0.001) in comparison with the HFD group. Interestingly, the ALP levels reached the control levels after the treatment. The levels of GGT also showed depletion in both treatment groups in comparison with the HFD group; however, the difference was not statistically significant. No significant difference was recognised in the bilirubin between different groups. But, the total bilirubin decreased in both treatment groups the difference was not significant. The statistical insignificance in some results could be explained by the small number of samples; however, it should be confirmed by future studies with large sample numbers.

## Conclusion

According to the obtained results, it is concluded that the *Origanum majorana* can protect the liver from oxidative stress-induced NFLD. This protective effect is related to the anti-oxidant compounds such as flavonoids, anthocyanins, tannin derivatives and rosmarinic acid. However, considering some statistically nonsignificant results, more studies are needed to confirm these results in a larger sample of rats. Also, further investigations could shed light on the exact role of oxidative stress in the pathogenesis of the disease and the effect of herbal medicine on this phenomenon. In this way, recruiting these medicinal plants in a therapeutic regimen may reduce the deleterious side effects of chemical agents.

## Figures and Tables

**Table 1:** Effect of HAEM on the serum lipid profile and liver enzymes in HFD-treated rats.

	NC group	HFD group	HFD + HAEM (150 mg/kg/day)	HFD + HAEM (450 mg/kg/day)
TG (mg/dL)	79.8(4.9)	95.8(5.0)*	53.4(14.3)###	36.6(10.2)###
Cholesterol (mg/dL)	76(7.3)	83.2(5.5)	77.6(9.6)	71.2(7.9)
LDL (mg/dL)	15.8(3.5)	29.8(5.1)	19.2(2.9)	21.4(4.9)
Bilirubin (mg/dL)	0.2(0.0)	0.26(0.04)	0.22(0.04)	0.22(0.04)
AST(IU/L)	196(33.9)	414(57.9)***	201(52.7)###	236.2(30.3)###
ALT(IU/L)	76.68(8.9)	224.6(62.6)	148.2(29.5)	134.6(37.7)
ALP(IU/L)	266.4(46.2)	422.4(58.0)***	256.4(38.0)###	240(19.2)###
GGT(IU/L)	1(0.6)	2(0.9)	1.2(0.4)	1.4(0.5)

Notes: Values are expressed as mean (standard deviation);

* *P* < 0.050;

** *P* < 0.010;

*** *P* < 0.001 compared to control;

# *P* < 0.050;

## *P* < 0.010;

### *P* < 0.001 compared to HFD-treated group

TG: F_(3,16)_=7.8, *P* < 0.001; Cholesterol: F_(3,16)_=0.4, *P* > 0.05; LDL: F_(3,16)_=2.0, *P* > 0.05; Bilirubin: F_(3,16)_=0.5, *P* > 0.05; AST: F_(3,16)_=5.1, *P* < 0.01; ALT: F_(3,16)_=2.3, *P* > 0.05; ALP: F_(3,16)_=3.9, *P* < 0.05; GGT: F_(3,16)_=0.4, *P* > 0.05
